# Design of a Low-Cost Gateway with LoRa Technology Serving Multiple Devices

**DOI:** 10.3390/s25164948

**Published:** 2025-08-10

**Authors:** Wuigor I. S. Bine, Linnyer B. R. Aylon

**Affiliations:** 1 Electrical Engineering Department, Federal University of Minas Gerais, Belo Horizonte 31270-901, Brazil; 2 Manna Research Group, State University of Maringa, Maringá 87020-900, Brazil

**Keywords:** LoRa, LoRaWAN, gateway, dual-channel, scalability, IoT

## Abstract

The growing demand for scalability and efficiency in Low Power Wide Area Networks (LPWANs) presents significant challenges, particularly due to the increasing number of connected devices and the inherent limitations of the ALOHA protocol, which is widely used in LoRaWAN networks. In this context, this work proposes the design and development of a low-cost dual-channel gateway tailored for Internet of Things (IoT) networks based on LoRa technology. To address the aforementioned challenges, this study explores approaches such as channel activity detection (CAD) and dynamic channel allocation, aiming to reduce collisions and optimize spectrum utilization. Experimental tests were conducted in environments subject to interference from coexisting networks to evaluate the performance of the proposed gateway. The results demonstrated significant improvements in packet delivery rate (PDR) and loss reduction, with PDR exceeding 94% for spreading factors (SFs) ranging from SF7 to SF12. In comparison, the single-channel gateway operating under the same conditions achieved a PDR between 80% and 85%. These results highlight the feasibility of the dual-channel gateway for small- and medium-scale IoT applications in scenarios with multiple coexisting networks.

## 1. Introduction

The IoT paradigm has been growing exponentially and brings with it challenges such as the increasing number of connected devices and the massive volume of data they generate, which are becoming critical issues for the scalability of IoT networks [1]. LPWANs have emerged as a promising alternative for large-scale IoT applications, in which devices distributed over vast areas transmit small data packets. The main LPWAN technologies include LoRaWAN, NB-IoT, Sigfox, and LTE-M [2].

LoRa technology, one of the most widely adopted in the LPWAN ecosystem, employs Chirp Spread Spectrum (CSS) modulation and operates in the sub-GHz ISM band, offering long-range connectivity, low power consumption, and affordable hardware costs [3]. However, an existing limitation of current LoRaWAN networks is their reliance on gateways that, in many cases, use hardware similar to the ones of end nodes, potentially restricting network scalability and performance [4].

The main limitation of LoRaWAN networks is related to the medium access protocol used, namely ALOHA. This protocol allows devices to transmit packets without performing prior channel sensing, which can lead to high collision rates in scenarios with a high density of nodes. Although it is widely adopted due to its simplicity of implementation and low energy consumption, the ALOHA model exhibits low spectral efficiency and limited scalability as the number of connected devices increases significantly [5,6,7].

In order to mitigate these limitations, the literature has explored several strategies, among which the use of the CAD mechanism stands out. CAD enables devices to check the channel status before initiating a transmission, functioning as a form of physical medium sensing. Its integration with multiple access protocols has proven effective in reducing collisions, increasing network efficiency, and saving energy in end devices by avoiding unnecessary retransmissions [8].

Significant efforts in academia have been dedicated to improving the efficiency of LoRaWAN networks [2,8,9]. The most studied approaches comprehend the optimization of ALOHA protocol variants, which aim to enhance channel utilization efficiency and reduce collisions, particularly in densely populated networks [5,10,11].

Channel activity detection (CAD) has emerged as an effective strategy, enabling devices to monitor the channel’s status prior to transmission, thereby significantly reducing collisions and improving overall network performance [8,12,13]. Complementarily, channel resource scheduling appears as a solution to allocate communication resources in a controlled manner, preventing interference and ensuring stable transmissions even in high-density scenarios [14,15,16].

Despite advances in research focused on optimizing the ALOHA protocol, implementing CAD, and scheduling channel resources, many applications still rely on gateways with hardware identical to that of sensor nodes [4,17,18]. This reliance is primarily due to the high cost of acquiring higher-capacity gateways. In these solutions, gateways typically integrate microcontrollers (MCUs) as the central control unit, responsible for network management and data storage, as well as providing remote access [19]. However, their radio frequency (RF) design generally includes transceivers with more limited features, which compromises performance compared to larger, higher-capacity gateways.

Given the growing demand for greater efficiency and scalability in LoRaWAN networks, implementing CAD mechanisms becomes essential in both gateways and end devices. CAD plays a critical role in optimizing channel access, serving as an initial step toward the adoption of more advanced protocols, such as Carrier Sense Multiple Access (CSMA), which enhances spectrum efficiency by reducing collisions during data transmission [2].

Although the literature presents several strategies to optimize communication in dense LoRa-based networks, including adaptive channel allocation [7], medium access control protocols such as Carrier Sense Multiple Access (CSMA) and Time Division Multiple Access (TDMA) [4], and collision avoidance mechanisms [9], fundamental limitations still persist. The development of LoRaWAN gateways often relies on more robust hardware platforms or commercial high-performance gateways to support the execution of complex algorithms [20]. In contrast, the proposed gateway was designed using low-cost hardware and supports local processing through a microprocessor capable of executing lightweight AI algorithms, making it suitable for resource-constrained environments.

Second, a significant portion of the literature focuses on single-channel architectures [20,21,22], which limits the simultaneous reception of data. The proposed model overcomes this limitation by incorporating multiple LoRa transceivers, enabling concurrent reception on distinct channels, thereby increasing throughput and reducing packet collisions. Third, in these single-channel gateway solutions, the CAD mechanism is typically implemented using a single transceiver responsible for both channel sensing and data reception [20,22]. This configuration may result in packet loss when the transceiver is performing CAD while a packet is being transmitted to the gateway, as it is unable to receive incoming packets during the sensing period.

To overcome this limitation, the proposed gateway dedicates one transceiver exclusively to CAD operations, ensuring that the reception channels remain continuously available. Finally, it is essential to consider hardware limitations related to time synchronization and data scheduling when designing IoT architectures. In this context, the proposed solution incorporates a GPS module and an RTC with TCXO, which together provide highly accurate timing even in the absence of Internet connectivity. This approach enables precise scheduling and improved coordination among devices, mitigating potential issues related to temporal misalignment in distributed networks.

In this context, this study aims to propose a low-cost hardware model for gateways operating with long-range technologies. The main contributions of this study are as follows: (i) the presentation of a dual-channel hardware model for low-cost LoRa technology gateways; (ii) the investigation of strategies to enhance LoRa network scalability through CAD in the proposed hardware model; and (iii) the discussion of the primary challenges and opportunities for IoT applications in urban and rural environments.

The remainder of this work is organized as follows: Section 2 presents the main aspects of LoRa technology and the LoRaWAN protocol; Section 3 discusses the key related works found in the literature; Section 4 describes the proposed hardware model for a dual-channel gateway capable of enabling multiple simultaneous connections; Section 6 presents a real-world case study comparing the performance of a single-channel gateway with that of the proposed dual-channel gateway; Section 8 addresses the main issues and challenges associated with the proposal; and finally, Section 9 provides the concluding remarks.

## 2. Background

In this section, we present the key aspects of LoRa technology and the LoRaWAN protocol.

### 2.1. Synopsis of LoRa Technology

LoRa technology has been considered for use in various urban applications, such as smart cities, as well as in non-urban scenarios, including agribusiness. Numerous studies have begun to analyze the performance of LoRa technology [23,24,25], focusing on comparisons with other technologies in terms of energy consumption and data transfer rates.

The evaluations conducted in these studies [23,24,25] highlight the advantages of LoRa technology combined with its LoRaWAN communication protocol, particularly for low power consumption and long-range capabilities, though with relatively low data rates, which are sufficient for many monitoring applications. Despite its initial success, open issues remain regarding scalability, performance, and the maximum traffic load supported in large-scale LoRaWAN networks [5,6].

LoRa technology is a spread spectrum technique that uses chirp pulses (Chirp Spread Spectrum—CSS), with its physical layer (PHY) developed by Cycleo to achieve long-range communication with low power consumption [26]. In recent years, this data transmission technology has become one of the primary choices for long-distance networks and communication with devices in harsh environments [27].

LoRa technology operates in the sub-GHz ISM (Industrial, Scientific, and Medical) band, which has specific frequency ranges in each country or region. The CSS modulation method used by LoRa in the sub-GHz bands ensures high robustness against noise arising from multipath propagation interference [28].

The PHY layer is defined by three main parameters: SF (spreading factor), BW (bandwidth), and CR (Error Correction Rate). The configuration of these parameters determines the data transmission rate, directly influencing the communication range and the power consumption of the transceiver.

- SF—Spreading factor: This is strongly related to the time required to transmit a data packet. The higher the SF, the more chirps are needed to encode a single bit in the modulation. Higher SF values increase the signal-to-noise ratio (SNR) [26,29].
- BW—Bandwidth: There are three predominant bandwidths used for transmission: 125, 250, and 500 KHz. The selected frequency range will be the one over which the LoRa chirp spreads. The higher the bandwidth, the greater the data rate of the packets; consequently, the transmission range decreases [26,29].
- CR—Error Correction Rate: The value set for CR corresponds to the number of bits added to the packet header to perform error correction techniques. When higher coding rates are defined, their robustness to interference significantly increases; however, the packet length increases along with the transmission time and power consumption [26,30].

The SF, configurable between 6 and 12, defines how many chips are used per symbol. According to the datasheet of the SX1276 transceiver [29], the higher the SF, the greater the receiver’s sensitivity, which increases the communication range. Conversely, the transmission time of the data will also be longer.

**Figure 1:**
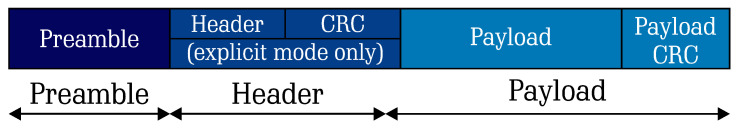
Typical structure of a LoRa packet, consisting of the preamble, header, payload, and CRC sections.

Figure 1 presents the standard structure of a transmission packet from a LoRa transceiver, which can be divided into a preamble, header, and payload. The preamble and header are configurable, allowing the explicit definition of the necessary configurations that the packet is sending to be detectable by the receiving side; it is also possible to know this information in advance, eliminating the need for it to be sent. Below, the three main characteristics of the packet structure are detailed.

- Preamble: The first part of the structure is used to synchronize the receiver with the data stream being transmitted through a sequence of symbols. For intensive reception, more symbols are required to reduce the duty cycle on the receiver, thus saving energy.
- Header: The header can be configured in an explicit or implicit manner. The explicit form transmits the encoding settings, payload size, and the presence of the cyclic redundancy check (CRC) field. However, the transmission time will be relatively longer compared to the implicit mode, which assumes these configurations are fixed and does not require their transmission.
- Payload: The payload is where the desired data is sent, encoded based on the error rate specified in the explicit header or the known rate when used in implicit mode. The length of the payload can be configurable, and an optional CRC can be added at the end. The amount of transmitted bytes influences the transmission time, along with the chosen SF for operation.

Despite the significant prominence of LoRa technology in monitoring applications and real-time data transmission, it faces challenges when applied in larger-scale networks, particularly in managing a high number of devices. Aspects such as scalability and maximum traffic capacity represent opened challenges in this research area.

### 2.2. Synopsis of the LoRaWAN Protocol

The LoRaWAN protocol is applied at the layer above the physical layer, known as the MAC (Media Access Control) layer, and was designed with the characteristics of LoRa technology in mind, as provided by the LoRa Alliance [31]. The use of this protocol has been gaining significant attention due to its features, which make it particularly suitable for IoT applications [26].

**Figure 2:**
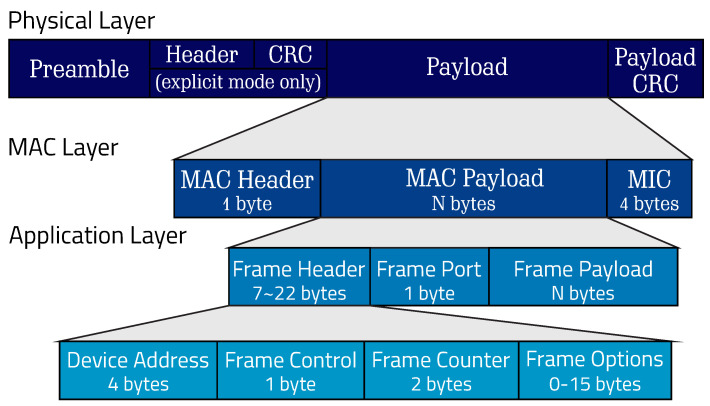
LoRaWAN network architecture highlighting the structure of the physical, MAC, and application layers, along with the composition of a typical LoRa packet.

The LoRaWAN protocol has a payload length limit that varies according to the selected SF and BW configuration, and it also considers whether MAC commands need to be transmitted within the MAC Payload [31,32]. Figure 2 shows the structure of the LoRaWAN protocol, which is transmitted within the physical layer’s Payload.

Observing Figure 2, the MAC layer is subdivided into MAC Header, MAC Payload, and MIC (Message Integrity Code). The MAC Header is used to define the protocol version, followed by the message type, which is used for management purposes. The MAC Payload is used to send the desired data and messages for network join requests or acceptances. The MIC is used to prevent message forgery and utilizes the MAC Header and MAC Payload to calculate its value.

The application layer is organized into three parts: the Frame Header, Frame Port, and Frame Payload. The Frame Header can contain from 7 to 22 bytes, where the device address and other control data are added. The Frame Port is determined based on the type of application and generally can range from 1 to 223, with 224 reserved for testing purposes [31]. Lastly, the Frame Payload carries the application data, which is encrypted with an application session key, typically following the AES 128 algorithm.

The size of the payload available for data transmission can be significantly reduced when utilizing all the available functions of the protocol. The maximum length of the payload for data transmission, excluding MAC commands, is 51, 115, or 242 bytes, depending on the bandwidth and SF used [32].

The network that utilizes LoRaWAN must have a star topology, and all sensor nodes support bidirectional communication, but it generally focuses on uplink transmission [33]. Thus, LoRaWAN networks consist of three main elements: (i) sensor nodes, capable of collecting data or activating actuators; (ii) gateways, which serve to provide connectivity between sensor nodes, acting as a bridge for the data path between devices and cloud services; and (iii) a network server, which manages the network, rejecting corrupted packets and scheduling messages to be transmitted to specific devices [26].

Starting from version 1.0.2 of LoRaWAN, terminal devices, or nodes, have been categorized into three classes: A, B, and C [31].

- Class A: This encompasses sensors that have the ability to receive data in predetermined time windows, immediately after performing a transmission. This class is characterized by supporting bidirectional communication.
- Class B: This includes actuators, also with bidirectional communication, but with scheduled reception windows.
- Class C: This encompasses devices that remain always available to receive data from the gateway or transmit information, offering greater communication availability.

In networks with a high density of devices, the need arises for more efficient gateways that can optimally manage the bidirectional communication of different classes of devices (A, B, and C). Class A devices, for example, open reception windows immediately after transmission, which requires a quick response from the gateway. However, in single-channel gateways using the ALOHA protocol, it may occur that, at the moment of transmission, a terminal device sends a message simultaneously. This can result in packet collisions or message loss, compromising the efficiency and reliability of the network.

## 3. Related Work

To address these issues, various efforts have been presented in the literature. Table 1 summarizes and compares existing studies proposing solutions for dense networks using LoRa technology, aiming to optimize the ALOHA protocol and utilize CSMA and TDMA to enhance performance and efficiency.

Several studies, such as those exploring TDMA and CSMA, focus on collision prevention, a significant challenge in dense networks. Approaches involving neighbor node listening [9] or enhancements to CSMA protocols [2,8,35] aim to minimize collision probability, thereby increasing communication reliability.

As presented in Table 1, several efforts have focused on software-based solutions aimed at improving communication and scalability in networks through advanced algorithms and protocols. These approaches highlight the importance of software level optimizations in addressing the challenges posed by the density of LoRa networks. However, many IoT devices face hardware limitations, and numerous applications are subject to cost constraints, making the development of smaller and more cost-effective networks a necessary alternative.

The literature shows that, in several proposed solutions, both end devices and gateways are often implemented using the same hardware [4,17,18]. Furthermore, studies focusing on software optimizations frequently address single-channel networks [2,8,20,22]. In such cases, it is common for the application of algorithms to assume the presence of robust gateways; however, this is not always the case, as seen in solutions that rely on identical hardware for both devices and gateways.

Several studies consider the use of multiple channels to reduce interference and increase network capacity. Adaptive use of multiple channels has proven effective in high-density scenarios, as shown in studies applying machine learning for channel selection [36] or using channel allocation algorithms [4,7].

Based on the studies analyzed, four main recurring challenges can be identified in LoRaWAN-based networks, particularly in scenarios with high device density:

- Scalability and efficiency in dense networks: The growing number of IoT devices imposes scalability challenges on LoRaWAN networks, requiring more robust mechanisms for medium access and resource management.
- Limitations of the ALOHA protocol: The use of pure ALOHA, without coordination among devices, results in high collision rates and packet loss, compromising communication efficiency.
- Lack of effective channel detection mechanisms: In low-cost implementations, it is common for gateways to use hardware identical to that of end devices. Even with the adoption of the CAD mechanism at the gateway, hardware limitations can compromise network performance. For instance, while performing CAD to send a response packet to a device, the gateway may miss simultaneous transmissions from other nodes, as it lacks the capability for parallel reception across multiple channels or instances.
- Need for low-cost multi-channel gateways: Most high-performance gateway solutions rely on specialized hardware, which are often expensive. In contrast, smaller networks or cost-sensitive applications frequently adopt gateways with hardware similar to that of sensor nodes, which significantly limits simultaneous reception capacity and reduces overall network robustness.

These challenges call for more accessible and efficient solutions, such as the one proposed in this work, which aims to combine multiple reception channels and CAD mechanisms in a low-cost gateway, contributing to the advancement of LoRaWAN networks across diverse application contexts.

## 4. A Dual-Channel Intelligent Gateway Model

In light of the challenges posed by the increasing number of connected devices in IoT networks and the need to improve communication efficiency, many efforts have been directed toward the development of solutions that optimize channel usage and reduce collisions.

LoRaWAN, which extensively utilizes the ALOHA protocol in conjunction with CAD mechanisms, has been explored to enhance the scalability of these networks. Furthermore, resource scheduling methods, such as TDMA, are applied to organize traffic and avoid interference, ensuring more efficient performance [2].

A promising solution to address these challenges is the implementation of dual-channel gateways, which allow simultaneous communication on two channels using LoRa transceivers. These gateways provide a cost-effective alternative to conventional eight-channel models, where the high cost can be a barrier for small- and medium-sized IoT networks. Although dual-channel gateways have some limitations in networks with a high density of devices, they are efficient in supporting multiple devices in smaller networks. Furthermore, the integration of CAD allows for the verification of channel occupancy before transmitting return data to devices, minimizing collisions and optimizing spectrum usage.

**Figure 3:**
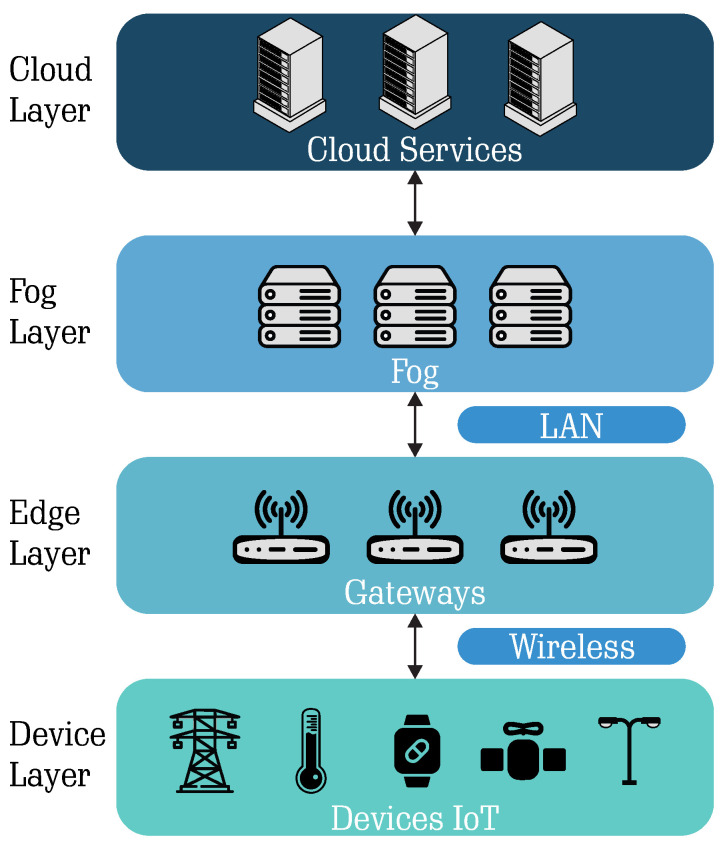
Layered IoT network architecture: Connected devices (Device Layer) communicate with gateways at the edge (Edge Layer), which interact with the fog (Fog Layer) and the cloud (Cloud Layer) for processing, analysis, and distributed services.

The architecture of IoT networks is illustrated in Figure 3, which describes the different layers of communication and processing. In the Device Layer, there are IoT devices that perform data collection or actions, while the Edge Layer is where the gateways are located, responsible for mediating communication between the devices and the next layer, the Fog Layer, which performs intermediate processing before sending data to the Cloud Layer, where the data is stored and processed.

**Figure 4:**
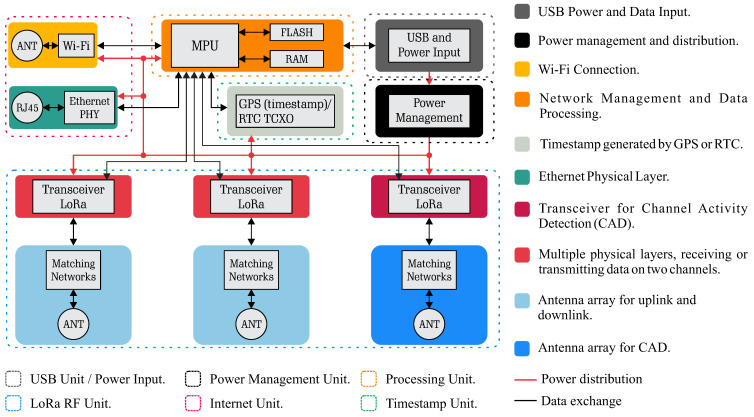
Proposed solution for a low-cost dual-channel LoRa gateway, highlighting the main functional blocks: processing unit, three LoRa transceivers (two for simultaneous reception and one dedicated to CAD), power management unit, time synchronization via GPS/RTC+TCXO, and connectivity through Ethernet and Wi-Fi.

The proposed low-cost dual-channel gateway is represented in Figure 4, implemented in the Edge Layer. These gateways are designed to provide simultaneous connections to multiple devices and can perform data preprocessing, enhancing the efficiency of the network.

Various solutions, as seen in [4,17,18], implement conventional gateways, where the transceiver responsible for data transfer typically can transmit to only one device at a time. In networks with many devices, this traditional method may not be the most suitable, leading to the need for solutions such as the dual-channel gateway, which enhances the capacity for efficient communication and management of multiple devices.

In this context, we observe a gap regarding the implementation of low-cost solutions for long-distance transmission using LoRa technology, particularly in small- and medium-sized networks. The model depicted in Figure 4 proposes the use of three LoRa technology transceivers (for example, SX127x or SX126x) in the RF unit, with two operating as transmitters and receivers on distinct channels and one specifically designated for implementing the CAD mechanism.

By proposing a set of antennas and a transceiver dedicated exclusively to the channel activity detection process, it ensures that the two primary channels remain available for data reception. Simultaneously, the channel dedicated to CAD monitors spectrum occupancy, ensuring that when it is necessary to transmit back to a device in the network (for example, during the reception window of Class A devices), the transmission occurs without interference. This avoids collisions and guarantees that the gateway does not lose information from other devices that may be transmitting at the time the return would be sent, thereby preserving the integrity of communication and the performance of the network.

The processing unit shown in Figure 4 is composed of a microprocessor, RAM, and FLASH memory, designed to enable processing, network management, and data storage. Thus, the microprocessor is relatively more robust than the microcontrollers found in the vast majority of IoT devices.

The transmission of data to the third or fourth layer (Figure 3) is enabled by the connectivity unit shown in Figure 4, which provides Internet access through various connection options, including wireless technologies such as Wi-Fi and mobile networks (3G/4G/5G), as well as wired connections via Ethernet.

The timestamp unit generates an accurate timestamp using GPS or a temperature-compensated crystal oscillator (RTC TCXO), ensuring proper synchronization, if necessary, between devices even without an Internet connection.

The power management unit is responsible for regulating the voltage to the other units, ensuring efficient management of the gateway’s energy consumption. The power input can be supplied through a USB connector, a dedicated power plug, or via PoE (Power over Ethernet).

It is important to note that the network management and data processing unit, by providing cloud services at the edge, enables meeting the latency requirements of applications, improving scalability and energy efficiency [19]. Furthermore, this approach offers the advantage of providing scalable services for delay-tolerant IoT applications that utilize LoRa technology.

In order to ensure that the design decisions and technical requirements of the system are aligned, Table 2 presents the main requirements considered in the development of the proposed model illustrated in Figure 4, organized into hardware and software categories. Each requirement is directly associated with the system’s operational needs, taking into account factors such as cost, simultaneous reception capability, scalability, and robustness in environments with interference from coexisting networks.

The main highlighted requirements include the capability for simultaneous reception on multiple channels and the execution of CAD without compromising the continuous listening of the dedicated transceivers for reception. To meet these requirements, the proposed model adopts multiple transceivers from the SX126x family, with two allocated for reception on distinct channels and a third dedicated exclusively to performing CAD.

To ensure accurate timestamping and message scheduling, the use of a GPS module in conjunction with a RTC+TCXO is proposed, particularly in situations where an Internet connection is unavailable. The processing unit must have sufficient capacity to efficiently manage the network and execute lightweight artificial intelligence algorithms, while meeting low-cost requirements.

In summary, the proposed dual-channel gateway provides a cost-effective solution for small- and medium-sized IoT networks, directly addressing the scalability and cost limitations of conventional solutions, such as large-scale gateways, as well as the constraints of single-channel gateways. With the integration of transceivers dedicated to channel activity detection and the ability to operate simultaneously across multiple channels, the model optimizes spectrum usage, minimizes collisions, and enhances the overall efficiency of the network.

## 5. Dual-Channel Gateway Applications

This section discusses the main application areas for dual-channel gateways based on LoRa technology, aiming to enhance the efficiency of small- and medium-scale networks while preserving the low-cost characteristic.

### 5.1. Smart Cities

Population growth in large urban centers has intensified social and economic challenges, such as traffic congestion due to increased vehicular flow, public transportation overload, environmental and noise pollution, inefficient solid waste management, failures in basic sanitation, and public safety issues. In this context, the IoT emerges as a strategic ally in the development of technological solutions that foster smarter and more sustainable cities, contributing to improved quality of life for the population.

Governments and institutions have been investing in the implementation of IoT-based solutions to enable the development of smart cities. However, the increasing density of connected devices imposes significant challenges on communication infrastructure, particularly with regard to scalability, energy efficiency, and data delivery reliability [1]. In LPWANs, such as those based on LoRaWAN, these limitations become even more critical. Although large-scale commercial gateways capable of simultaneous multi-channel reception are available, their high cost remains a barrier to the deployment of applications with budget constraints.

The use of dual-channel gateways emerges as an intermediate and cost-effective solution, offering significant performance gains over single-channel gateways while maintaining substantially lower costs compared to more robust commercial models. This approach enhances network capacity by reducing collisions and improving the packet delivery rate, thereby enabling the deployment of scalable and efficient IoT systems in urban environments.

#### 5.1.1. Smart Metering

Applications that require consumption monitoring and management can implement smart metering to collect such metrics. The most common use cases include electricity, water, and gas consumption; however, in industrial settings, additional possibilities arise, such as the measurement of oils, seeds, grains, and flours in storage tanks [1].

**Figure 5:**
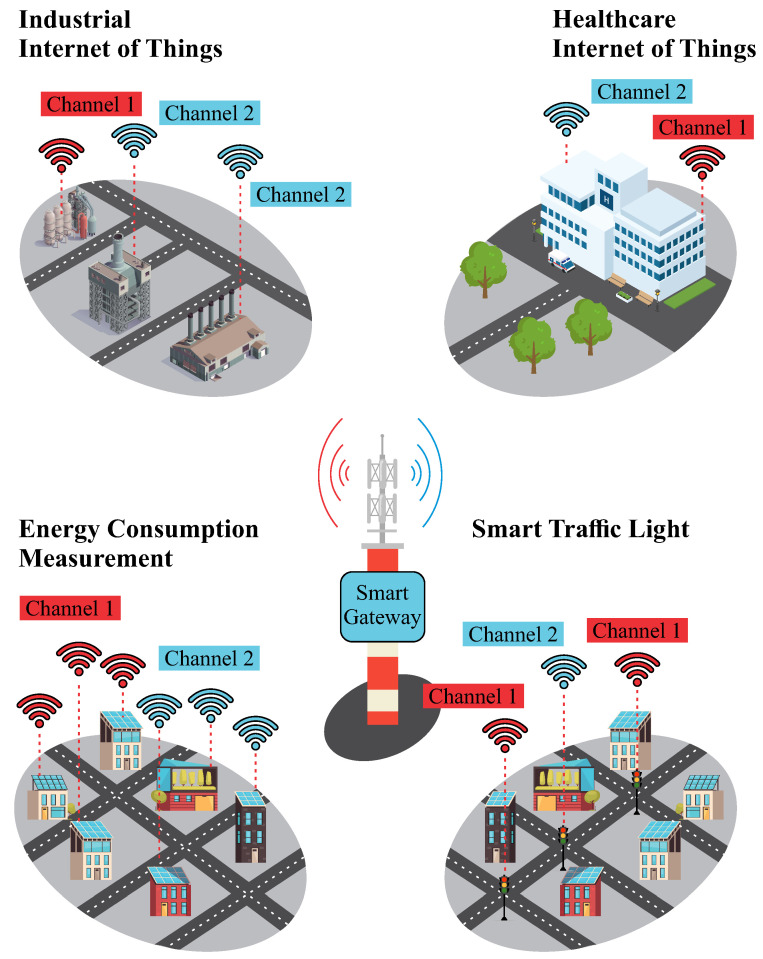
Dual-channel long-range gateway application, exemplified by smart meters. The architecture illustrates the logical separation of devices into two communication groups (Channel 1 and Channel 2), enabling simultaneous reception, reducing collisions, and improving efficiency in high-density deployment scenarios.

Figure 5 illustrates the use of smart meters as multiple nodes connected to the network, which transmit consumption data to utility companies. In this scenario, the use of dual-channel gateways enables simultaneous communication with multiple devices, even in regions with high sensor node density. This capability allows, for example, the urban infrastructure to be segmented into zones or neighborhoods, with each group of meters operating on distinct channels, thereby reducing collisions, optimizing spectrum usage, and lowering infrastructure costs.

In addition, the dual-channel gateway provides greater real-time reception capacity, enabling utility companies to monitor consumption patterns more effectively. Urban centers also contain numerous sensitive areas, such as regions with factories and industries handling chemical products, as well as nuclear power plants, which pose potential risks to the population. Monitoring these environments is therefore essential [1].

#### 5.1.2. Smart Traffic Light

The use of smart gateways on urban roads plays an important role in improving vehicle traffic by making roadways more efficient through vehicle flow monitoring and traffic light control. These systems can work in conjunction with artificial intelligence applications to redirect traffic in the event of accidents or even to clear lanes for the passage of special vehicles, such as ambulances and police cars.

Although it may appear to be a simplistic application, the use of smart traffic lights must be stable and reliable. The desynchronization of a single unit due to communication failure can lead to traffic congestion and hinder mobility on roads in large urban centers.

In densely built urban environments or regions with a high concentration of vegetation surrounding traffic lights, the use of multiple gateways becomes necessary to ensure adequate coverage and reliable communication between devices. In this scenario, the adoption of dual-channel gateways represents a cost-effective alternative compared to large-scale commercial gateways.

#### 5.1.3. Healthcare Internet of Things (HIoT)

Reliable transmission of health data is essential for the proper functioning of medical networks based on connected devices, such as wearables, vital sign sensors, and remote monitoring systems [37]. In hospital environments, where multiple patients are monitored simultaneously, network responsiveness becomes critical, especially in situations that require rapid decision-making and immediate intervention.

The use of dual-channel gateways in this context offers significant benefits, particularly in hospitals or healthcare units with a high density of connected devices. This architecture helps reduce the risk of collisions, increasing the reliability of communication between sensors and clinical systems. Furthermore, combining the dual-channel gateway with edge computing capabilities enables the execution of machine learning algorithms for local data analysis, contributing to early diagnosis, disease prevention, and support for physical rehabilitation programs, with faster and more intelligent responses [37].

#### 5.1.4. Industrial Internet of Things (IIoT)

Among the numerous possibilities introduced by the IoT paradigm is its application in industrial environments. The increasing capability of sensors to perform local processing, albeit still with certain limitations, combined with emerging communication technologies, has led to significant growth in the deployment of IIoT devices.

The demand for wireless devices in IIoT has grown considerably, driven by the need for more robust hardware solutions with lower susceptibility to electromagnetic noise and greater operational reliability, as provided by LoRa technology [37].

In large-scale industrial plants, it is common to find numerous connected devices dedicated to predictive maintenance and continuous monitoring of production processes. The integration of the dual-channel gateway with artificial intelligence techniques contributes significantly to reducing operational costs and overall production time.

Early fault detection in machinery allows industries to avoid unexpected interruptions, which often result in significant financial losses. To enable such diagnostics, multiple sensor nodes are deployed on critical equipment to collect data that allow real-time assessment of the condition and remaining useful life of motors and other essential industrial components [38].

In addition to the high density of monitoring devices, traceability systems are also widely employed in industrial environments for asset, raw material, and product tracking using LoRa technology [38]. In such scenarios, the use of dual-channel gateways enables the efficient segmentation of devices across multiple communication channels, enhancing network scalability. This approach helps reduce collisions without requiring substantial investments in infrastructure.

### 5.2. Smart Agriculture

The adoption of technologies in agriculture has expanded rapidly in recent years, with the application of artificial intelligence techniques proving useful for optimizing agricultural processes [37]. Large cultivation areas typically include numerous sensors collecting environmental data, and many of these applications rely on cloud-based processing, which may result in significant response times. Another critical issue is the limited signal coverage from cellular providers in non-urban areas, making data exchange difficult or even unfeasible due to lack of connectivity.

**Figure 6:**
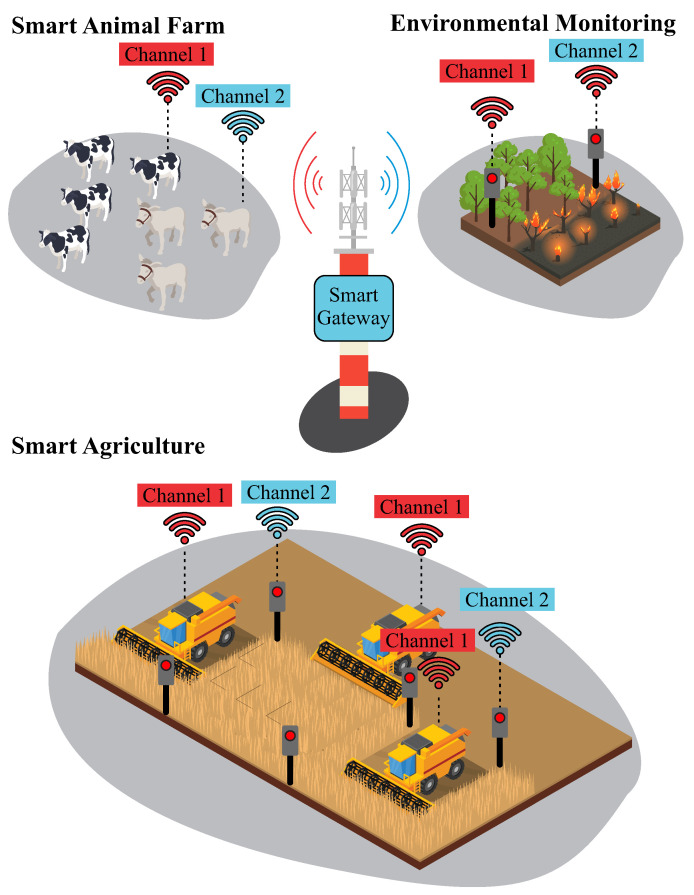
Applications of the long-range smart gateway in non-urban environments. The use of distinct channels (Channel 1 and Channel 2) enables simultaneous management of agricultural, livestock, and environmental sensor data, enhancing scalability and reducing collisions.

Figure 6 illustrates several applications that benefit from the adoption of dual-channel gateways, which, in addition to enabling long-range data transmission, represent an effective solution to the connectivity and scalability challenges faced in this sector. By allowing simultaneous data reception across multiple channels, this architecture promotes more robust and reliable communication between field-deployed sensors and control systems. This capability is particularly relevant in remote regions, where conventional communication infrastructure is limited or nonexistent, requiring solutions that operate autonomously and are resilient to the adverse conditions of rural environments.

#### 5.2.1. Smart Animal Farm

Soil monitoring to detect disturbances in its properties accelerates pasture recovery and supports animal management on large farms. Disease detection through data collection from animals assists in disease prevention, contributing to animal welfare.

The use of dual-channel gateways, combined with the application of artificial intelligence techniques, enables the execution of machine learning algorithms directly at the network edge. This approach allows for rapid response in critical decision-making processes, particularly in rural or remote environments where mobile network coverage is often unavailable or unstable. The ability to locally process and react to collected data reduces dependence on centralized infrastructure, increasing the autonomy and operational efficiency of agricultural applications.

The implementation cost of a long-range dual-channel gateway is relatively low, making it an economically viable alternative for distributed environments such as large agricultural areas. This solution reduces the number of required repeaters, as the gateway can be configured to operate in mesh architectures, thereby extending coverage and improving communication reliability. Compared to other technologies that require a significant number of repeaters to ensure signal propagation, the dual-channel gateway stands out for its efficiency in both operational and cost terms.

#### 5.2.2. Environmental Monitoring

Environmental monitoring applications based on IoT include wildfire detection, tracking of fauna and flora, identification of landslides in areas near highways and residential zones, pollution monitoring, and the detection of seismic events such as earthquakes and tsunamis [1].

These applications require a high level of communication reliability and security, as failures or vulnerabilities may lead to severe consequences. The generation of false positives, such as erroneous earthquake alarms, can result in substantial logistical and financial losses for public authorities. On the other hand, the failure to detect critical events may endanger human lives and lead to significant property losses [1].

Given the exposure of sensors to extreme conditions, particularly in natural disaster scenarios such as wildfires, these devices are commonly designed with limited computational resources, prioritizing robustness and energy efficiency. However, despite hardware constraints, these devices are equipped with high-precision sensors and mechanisms that ensure the integrity and security of the collected data, thereby guaranteeing the reliability of the information transmitted to the network.

In this context, the adoption of dual-channel gateways with long-range communication capability constitutes an architecture that not only enables the simultaneous reception of transmissions from multiple sensors but also allows the execution of computational tasks at the network edge. This significantly reduces latency and increases system resilience in critical situations. By combining parallel reception and local processing, dual-channel gateways contribute to a faster and more efficient response to extreme environmental events, ensuring greater robustness for distributed monitoring applications.

## 6. Case Studies

With the increasing popularity of IoT and devices that utilize LPWAN technologies such as LoRa, the deployment of new applications may take place in areas where private or public networks already exist, either nearby or at the same location.

To evaluate the performance of the dual-channel gateway, the design presented in Figure 4 was adopted as the foundation for the development and production of the prototype. The system architecture was designed using LoRa SX1262 transceivers, responsible for communication over two distinct channels, while the central processing unit was based on the ESP32-S3FH4R2 microcontroller, which integrates 4 MB of internal flash memory and 2 MB of internal RAM. Furthermore, the project allows for the expansion of storage and processing capabilities through the incorporation of external flash memory (MX66U1G45G, 128 MB) and additional RAM (APS6404L-3SQR, 8 MB), providing flexibility for more demanding applications while maintaining low prototyping costs.

The selection of the ESP32-S3 family is justified by the presence of a vector acceleration unit, designed to support applications with computational requirements associated with embedded artificial intelligence (AIoT). This feature enables the efficient execution of inference algorithms, such as convolutional neural networks (CNNs), directly on the device, eliminating the need for offloading to external servers. The ESP32-S3 natively supports machine learning frameworks optimized for embedded systems, such as TensorFlow Lite for Microcontrollers [39] and the Espressif ESP-DL library [40], allowing the integration of lightweight AI models for real-time analysis with low latency and reduced power consumption—key characteristics for edge computing.

**Figure 7:**
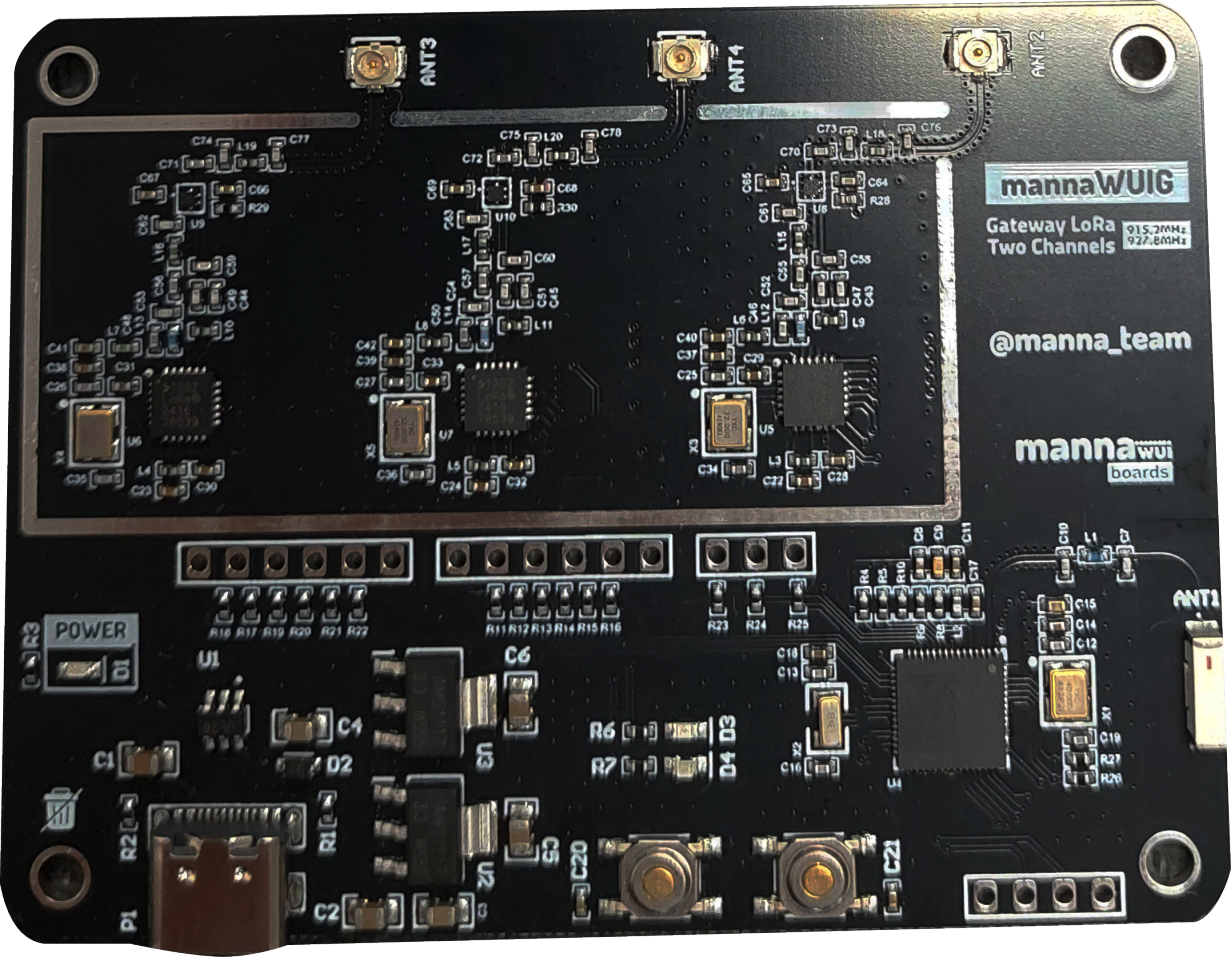
Final prototype of the dual-channel gateway developed for experimental testing.

The final version of the device, used in the experimental tests, is presented in Figure 7. The sensor nodes employed in this application correspond to an enhanced version of the device described in [41].

For comparative purposes, the application was initially developed using only sensor nodes, with one device, identical to a sensor node, configured to operate as a single-channel gateway. Subsequently, for each scenario, the same application was executed using the proposed dual-channel gateway. The ALOHA-based LoRaWAN protocol was adapted and employed across all scenarios. The experiments were conducted in a university environment with the presence of coexisting LoRa networks, which enabled the evaluation of the proposed gateway under real-world operating conditions.

**Figure 8:**
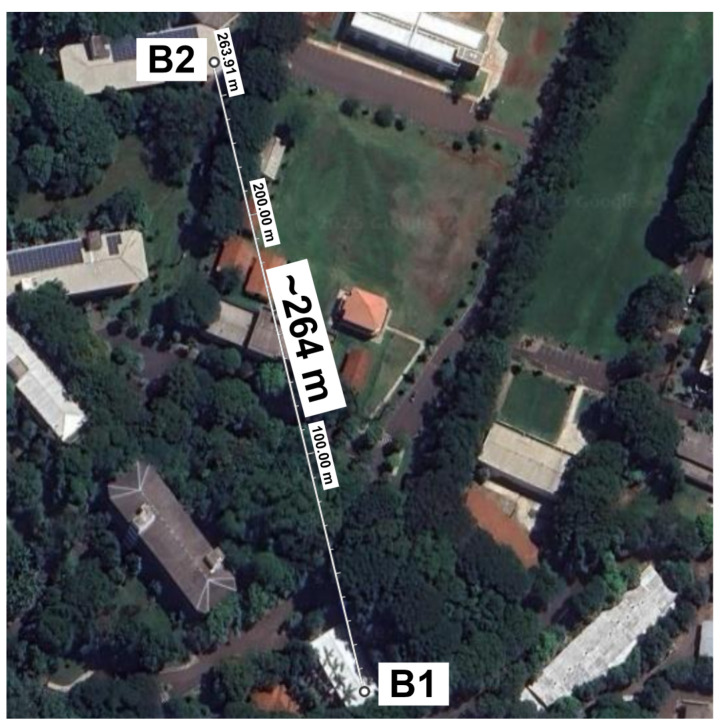
Location where the devices were installed, distributed between blocks B1 and B2.

Both gateways, single-channel and dual-channel, were installed in the research laboratory building. The sensor nodes were distributed across two university buildings, as shown in Figure 8, separated by a straight-line distance of approximately 264 m.

**Figure 9:**
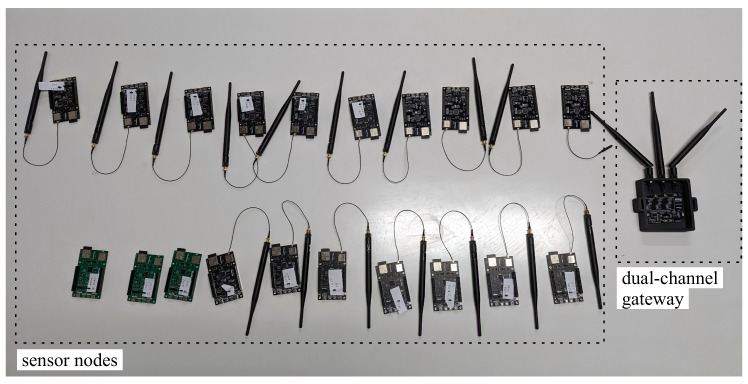
Sensor nodes used for testing and the dual-channel gateway.

Figure 9 shows the dual-channel gateway along with the 20 sensor nodes used for ambient temperature and humidity monitoring. The LoRa network was configured with the following parameters: a bandwidth (BW) of 125 kHz, a coding rate (CR) of 4/5, and a transmission power set to 10 dBm.

In all evaluated scenarios, the transmitted payload consisted of 4 bytes, representing temperature and humidity values. Considering the headers and commands of the LoRaWAN protocol, the total size of the transmitted packet was 17 bytes.

During the experimental period, weather conditions remained stable, with no rainfall events. The average maximum temperature recorded over the seven days of testing was approximately 29 °C.

### 6.1. Scenario 1

For the evaluation of the first scenario, the devices performed ambient temperature and humidity measurements, transmitting the data at intervals ranging from 1 to 2 min. For each transmission, the time was randomly selected using a fixed seed across all scenarios to ensure consistency in the results. The devices were configured to operate in Class A mode, opening up to two receive windows after each transmission in order to receive acknowledgment from the gateway.

The single-channel gateway randomly selects its transmission channel during the initial configuration phase and maintains it as fixed throughout the entire operation. In the case of the dual-channel gateway, as devices join the network, each one is assigned to one of the two available channels. The configuration information is transmitted via a downlink channel previously defined by the algorithm.

To ensure synchronization, the LoRaWAN protocol implemented on the sensor nodes was adapted so that each device transmits exclusively on the channel previously assigned by the gateway.

In this scenario, the two channels on which the gateway will operate are randomly selected during its initialization and configuration. From that point on, all devices joining the network operate within these two channels. For each newly integrated device, the gateway evaluates the distribution of devices across the channels and assigns the new device to the channel with fewer devices, aiming to maintain a balanced load between the two available channels.

### 6.2. Scenario 2

In this scenario, channel activity detection (CAD) was used prior to each transmission. The same configurations from the first scenario were maintained; however, the number of CAD checks performed before the actual data transmission was additionally recorded.

### 6.3. Scenario 3

In this scenario, the performance of the dual-channel gateway is evaluated while operating under a dynamic channel-switching policy based on time synchronization between the devices and the gateway.

In LoRaWAN networks, devices typically perform uplink transmissions on randomly selected channels from the 64 available. However, when using a gateway that supports only two channels, it becomes necessary to ensure that both the device and the gateway are operating on the same channel at the time of communication; otherwise, packet loss may occur.

To ensure this synchronization, a deterministic channel selection mechanism was implemented using a timestamp obtained during the network join process. Both the devices and the gateway use this timestamp, composed of the hour (*h*) and minute (*m*) variables, as the basis for calculating the active communication channel, as described in Equation (1).

(1) channel=((h×60)+m)×MOD(CH)

where *h* represents the current hour, *m* corresponds to the current minute, and *CH* denotes the number of available channels for uplink or downlink transmissions.

This mechanism ensures that, at each new minute, the transmission channel is switched in a synchronized manner between the device and the gateway, without the need for additional signaling. Since the calculation is based on a deterministic function shared by both ends, synchronization is maintained as long as the time base remains aligned.

To ensure temporal synchronization, both the gateway and the sensor devices are equipped with RTC+TCXO modules. The initial timestamp is provided by the gateway at the moment the device joins the network, and from that point on, each node maintains its local time count using the RTC+TCXO, ensuring the precision required for coordinated channel switching.

The use of all 64 available channels for LoRaWAN transmissions significantly contributes to mitigating congestion in environments with multiple coexisting networks. In scenarios where simple applications, often based on single-channel gateways, operate on fixed frequencies, there is a tendency for specific channels to become overloaded, which increases the likelihood of collisions.

In this context, the periodic and synchronized channel switching adopted by the proposed dual-channel gateway represents a strategy to more evenly distribute traffic across the spectrum. Although the gateway operates simultaneously on only two channels, the dynamic switching mechanism allows for better utilization of the available frequency diversity, reducing interference and increasing communication robustness, especially in networks with high device density or in environments with multiple coexisting LoRaWAN networks.

## 7. Results

This section presents the performance analysis of the developed dual-channel gateway, based on the previously described experimental scenarios. The objective is to demonstrate that the proposed low-cost solution delivers satisfactory performance in small- and medium-scale IoT networks.

To evaluate network performance in each scenario, the Packet Delivery Ratio (PDR) metric was used, representing the ratio between the number of successfully received packets and the total number of transmitted packets. The experiments were conducted over 7 consecutive days for each of the three evaluated scenarios, totaling 21 days of experimental data collection. In each scenario, each device transmitted an average of 6270 packets.

**Figure 10:**
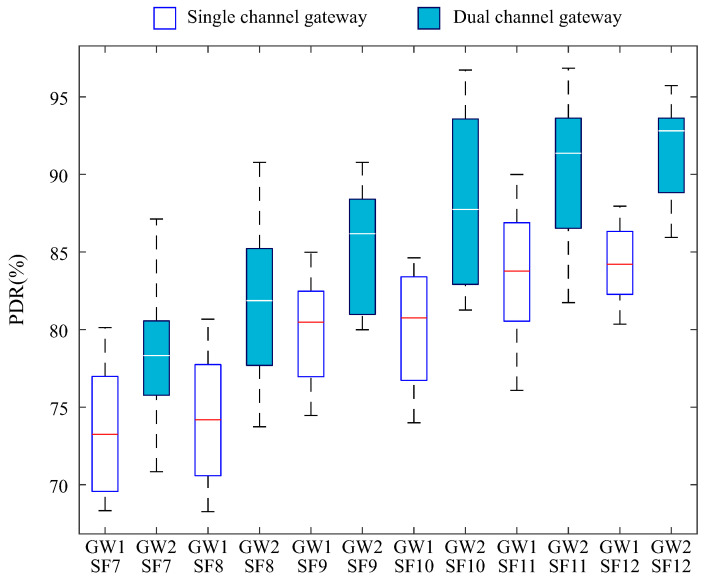
PDR segmented by spreading factor for the first scenario.

Figure 10 presents the PDR distribution for the first scenario, segmented by spreading factor (SF 7 to 12) for both types of gateways. GW1 corresponds to the single-channel gateway, while GW2 refers to the dual-channel gateway.

For all SF values, the dual-channel gateway achieved higher PDR values compared to the single-channel gateway, highlighting the greater communication efficiency when multiple reception channels are available.

The performance of GW1 at SFs 7 and 8 (Figure 10) shows lower medians, which is directly related to the limitations of the single-channel gateway and the use of a pure ALOHA-based LoRaWAN protocol. In this context, the lack of coordination among devices may lead to simultaneous transmissions, resulting in packet collisions.

It is observed that as the SF increases, the PDR improves for both gateways. However, the single-channel gateway exhibits greater variability. In contrast, the dual-channel gateway demonstrates increased consistency from SF9 onward, maintaining the PDR consistently above 85%.

The use of high spreading factors, such as SF11 or SF12, is associated with an increase in PDR due to greater signal robustness over long distances. However, this gain comes at the cost of longer transmission times, which increases energy consumption. Moreover, in scenarios with high device density, the extended channel occupancy time associated with high SFs may intensify the occurrence of collisions, compromising PDR performance [21].

The use of a dual-channel gateway offers the benefit of distributing devices across different channels, thereby reducing the density of concurrent transmissions on a single channel. This contributes to a decrease in the average channel occupancy time and, consequently, reduces the probability of collisions. On the other hand, this approach entails an increase in hardware cost due to the need to support multiple channels simultaneously when compared to single-channel gateways.

**Figure 11:**
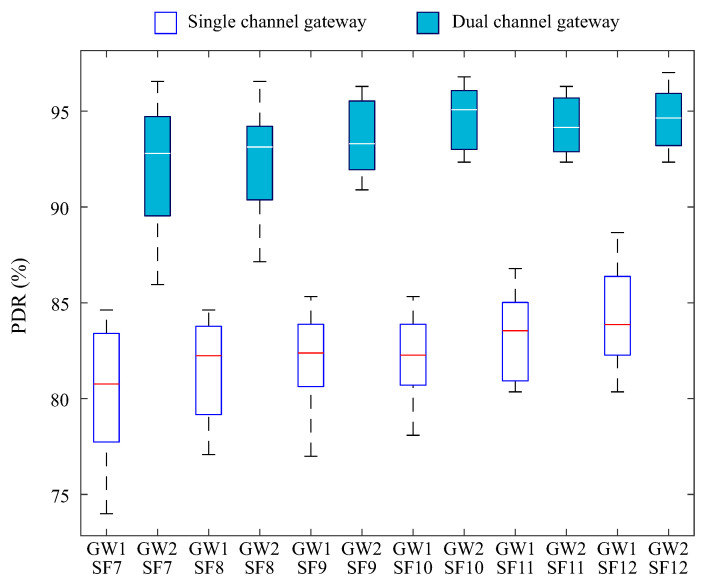
PDR segmented by dispersion factor for the second scenario.

The second scenario exhibits a more concentrated PDR compared to the first, indicating an improvement in the packet delivery rate. Figure 11 presents the PDR values for SFs 7 to 12, considering both gateways. An overall increase in PDR is observed compared to the first scenario, as a result of the application of the CAD mechanism, which helps prevent simultaneous transmissions and minimize collisions.

The adoption of the CAD mechanism resulted in a significant improvement in the performance of the single-channel gateway, with PDR medians exceeding 80% across all evaluated SFs. In the case of the dual-channel gateway, its inherent capability to operate over multiple channels already provides greater efficiency in packet reception. When combined with the application of CAD, this design demonstrated superior performance, with PDR medians exceeding 92%, standing out as a more efficient hardware design compared to the single-channel configuration.

This behavior is consistent with the literature, which demonstrates that the use of CAD significantly reduces the occurrence of collisions and also contributes to energy savings in end devices by avoiding unnecessary retransmissions and reducing transceiver activation time during packet transmission attempts [8].

In the third scenario, a channel-switching mechanism was introduced for uplink transmissions, utilizing the 64 available channels in order to optimize spectrum usage and reduce traffic concentration on a limited number of channels. This approach was combined with the use of CAD, which was already implemented in the second scenario.

**Figure 12:**
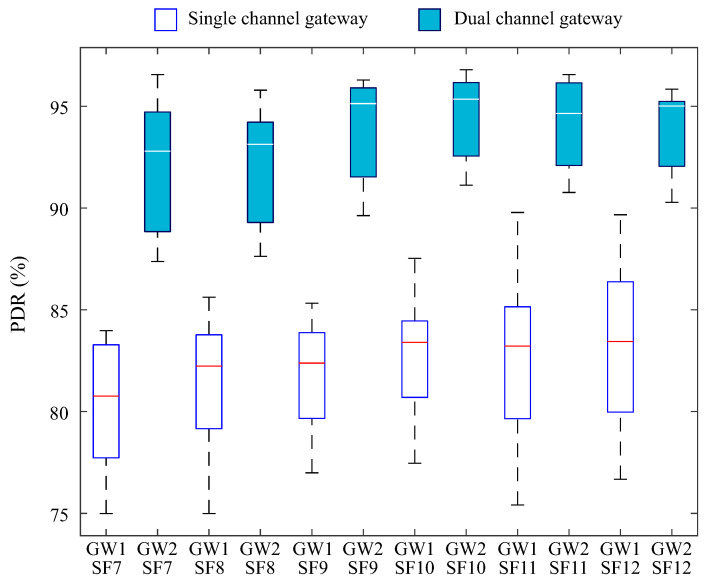
PDR segmented by dispersion factor for the third scenario.

Figure 12 shows a greater dispersion in PDR values among the devices when compared to the second scenario, in which the gateway’s two channels were randomly fixed during initialization.

The use of all 64 available uplink channels in environments with coexisting LoRa networks increases the likelihood that the dual-channel gateway will operate on a channel with a higher traffic load at certain times. This occurs when one or more devices from other networks transmit simultaneously on the same channel to which the gateway is tuned, increasing the probability of collisions and negatively impacting packet reception.

Despite a slight increase in data dispersion, the PDR medians for the dual-channel gateway showed improvement compared to the previous scenarios, reaching 96% for SF9, 95% for SF10, 94% for SF11, and 95% for SF12. The channel-switching strategy was also applied to the single-channel gateway. Compared to the second scenario, its PDR median remained between 80% and 85%.

It is also observed that in both scenarios, the increase in SF led to an improvement in PDR. This occurs because the number of symbols transmitted per bit increases exponentially with the SF, resulting in a longer transmission time for each packet [29]. This extension makes the signal more robust against interference and noise, as it increases the symbol spacing.

Although increasing the SF makes the signal more robust and less susceptible to interference, it does not guarantee a significant improvement in the performance of the single-channel gateway. At higher SFs, the channel occupancy time increases considerably, causing the gateway to spend more time receiving a single packet. As a result, the channel may be occupied when other devices attempt to transmit, increasing the likelihood of collisions and packet loss.

This phenomenon also occurs in the dual-channel gateway; however, the ability to operate simultaneously on two channels provides a significant advantage over the single-channel gateway. The parallel reception capability enables a more balanced distribution of transmissions across the available channels, reducing the risk of collisions even under high channel occupancy conditions. When combined with the CAD mechanism, this feature leads to improved network performance, as evidenced by the results presented.

**Figure 13:**
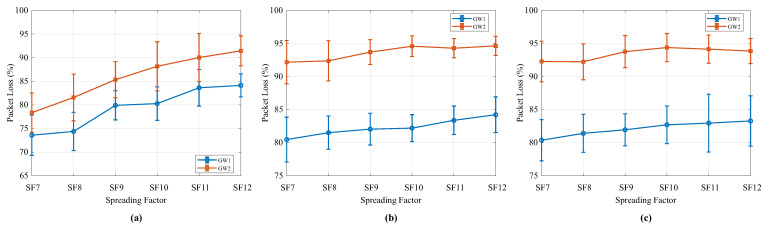
Interaction plots between gateway type, single-channel (GW1) and dual-channel (GW2), and spreading factor (SF7 to SF12) on packet loss rate (%) in the three experimental scenarios: (**a**) Scenario 1, (**b**) Scenario 2, and (**c**) Scenario 3.

Based on the results obtained across the different scenarios, a two-way analysis of variance (two-way ANOVA) was also performed. The results are illustrated in Figure 13, which presents the interaction plots between the Gateway and SF for the three analyzed scenarios: Scenario 1 (a), Scenario 2 (b), and Scenario 3 (c).

In all scenarios, the Gateway factor exhibited a statistically significant effect on packet loss: Scenario 1, F(1,228) = 154.77, *p* < 0.0001; Scenario 2, F(1,228) = 1321.11, *p* < 0.0001; and Scenario 3, F(1,228) = 916.17, *p* < 0.0001. These results indicate that the dual-channel gateway consistently outperformed the single-channel gateway in terms of packet delivery rate, regardless of the SF used.

The SF factor also had a significant influence in all cases: Scenario 1, F(5,228) = 55.02, *p* < 0.0001; Scenario 2, F(5,228) = 9.41, *p* < 0.0001; and Scenario 3, F(5,228) = 4.61, *p* = 0.0005. Packet loss tends to increase with higher SF values, which is consistent with LoRa modulation. Higher spreading factors result in longer transmission times due to the increased number of symbols, which may lead to missed receptions of other packets.

On the other hand, the interaction between the Gateway and SF factors was not statistically significant in any of the scenarios: Scenario 1, *p* = 0.4962; Scenario 2, *p* = 0.4835; and Scenario 3, *p* = 0.8716. This indicates that the effect of the gateway type is consistent across different SF values. The superior performance of the dual-channel gateway over the single-channel gateway is maintained regardless of the spreading factor used. This lack of interaction is also visually confirmed in the plots shown in Figure 13, where the lines representing the single-channel gateway (GW1) and the dual-channel gateway (GW2) remain approximately parallel across the different SF values.

The results show better performance for the dual-channel gateway across all scenarios. They also demonstrate that, although increasing the SF negatively impacts the packet delivery rate, this influence does not alter the relative advantage between the two evaluated gateways.

Table 3 presents the average number of channel activity detection (CAD) attempts required before each packet transmission to the dual-channel gateway, showing an average of 1.34 attempts in the second scenario and 1.51 in the third. It is observed that, in the third scenario, some devices required a higher number of CAD attempts.

This behavior is related to the channel-switching strategy adopted by the dual-channel gateway, which switches to a new channel every minute based on Equation (1). As a consequence, the gateway may, for certain intervals, operate on channels with higher occupancy due to coexisting networks, increasing the likelihood of the channel being busy and requiring more CAD checks before transmission.

The experiment was conducted within a university campus, where multiple research projects utilizing LoRa technology coexist. It is plausible that some of these networks employ single-channel gateways fixed on specific channels. Therefore, when the dual-channel gateway switches to a channel that coincides with one of these networks, an increase in the number of CAD attempts is observed, as seen in the third scenario, due to the temporary rise in channel occupancy.

**Figure 14:**
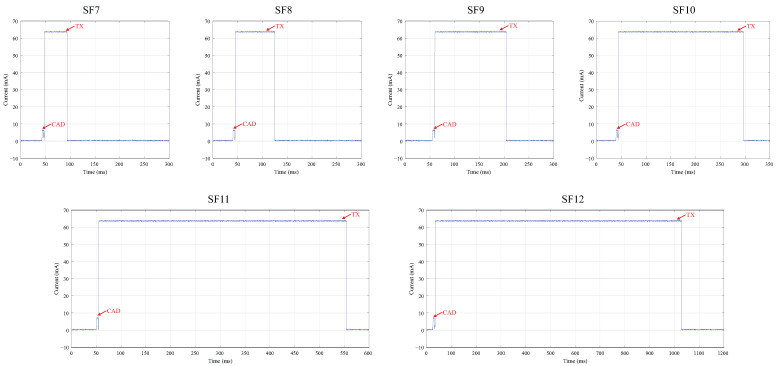
Current consumption during transmission for spreading factors SF7 to SF12.

In the case study, the devices were configured to transmit at a power level of 10 dBm, resulting in an approximate current consumption of 64 mA during transmission and 6.19 mA during the execution of the CAD preceding the transmission, as illustrated in Figure 14.

As the SF increases, the transceiver remains active for longer periods during transmission, resulting in higher energy consumption. This is a critical factor for battery-powered devices. In the context of using the dual-channel gateway in environments with coexisting LoRa networks, the channel-switching strategy implemented in the third experimental scenario aims to optimize the use of the available spectrum. Despite the need to perform a greater number of CAD checks before transmission, the energy consumption and duration of the channel detection procedure are significantly lower, as shown in Figure 14, when compared to the actual data transmission.

In the case studies presented, involving an application with 20 devices operating in environments with coexisting LoRa networks, the use of a single-channel gateway may limit the packet delivery rate to approximately 80% at the lower SFs.

Although increasing the SF may contribute to improved packet delivery rates, even for devices located near the gateway, this strategy results in significantly longer channel occupancy times. As illustrated in Figure 14, a transmission using SF12 lasts for nearly one second, which directly impacts the device’s energy consumption. By employing the proposed gateway design, it is possible to achieve an average delivery rate above 90% even at lower SFs.

The use of CAD helps reduce collisions; however, it does not guarantee that the channel will remain unoccupied at the exact moment transmission begins. This limitation becomes more evident in single-channel gateways, where the probability of collision is higher, especially as the number of devices in the network increases or in environments with coexisting LoRa networks.

The dual-channel gateway design proposed in this study presents an efficient alternative for small- to medium-scale networks, as it enables the simultaneous reception of packets on multiple channels while maintaining low implementation costs. This approach improves the packet delivery rate and contributes to reduced energy consumption in network devices.

## 8. Challenges and Important Issues

IoT devices have been widely employed to enhance user well-being, optimize business and industrial processes, increase security, promote mobility, and strengthen public health, among many other applications. The significant growth in the number of these devices, combined with the massive generation of data and the need for real-time responses, imposes new demands on network infrastructure. As more advanced gateways are developed, both in terms of hardware and software, with greater processing capacity and embedded functionalities, new requirements and challenges also emerge, such as the following.

### 8.1. Number of Devices

The growing number of intelligent applications across various contexts, including smart homes and buildings, markets, shopping centers, enterprises, industries, agriculture, and healthcare, has driven the increasing use of connected devices [9,37,42]. This progress intensifies the demand for hardware solutions, particularly gateways capable of supporting a large number of connections in small-, medium-, and large-scale networks, while remaining cost-effective. Although most studies focus on software-based approaches, the need for the development of dedicated hardware that meets these requirements and enhances the effectiveness of proposed solutions has become increasingly evident.

### 8.2. Mobility

The mobility of IoT devices connected to Wi-Fi or cellular networks (4G/5G) presents limitations, which justifies the adoption of LoRa technology in scenarios that require long-range communication. However, as the node moves farther from the gateway, it becomes necessary to adjust transmission parameters, such as increasing the spreading factor (SF), in order to ensure greater signal robustness. This adjustment results in longer transmission times, which poses a challenge for applications that demand low latency and timely decision-making, such as tractors and harvesters, whose distance from the gateway varies constantly during operation. To enable mobility in latency-sensitive applications within long-range networks, it is essential to migrate processing services to a smart gateway located close to the point of data generation. In this context, there is a growing need for the joint development of hardware and software solutions that enable edge processing, promoting greater autonomy and operational efficiency [43].

### 8.3. Centralized vs. Distributed Data Processing

The significant increase in the volume of data generated by IoT devices demands more efficient distributed processing solutions. Although the training of complex models remains predominantly concentrated in cloud infrastructures, there is a growing challenge in developing hardware with sufficient computational capacity for local inference execution [44].

In this context, adopting gateways capable of executing lightweight AI algorithms becomes a viable alternative. This approach enables distributed data processing at the network edge, reducing cloud dependency and promoting a balanced relationship between cost and computational capability.

### 8.4. Security and Privacy

The adoption of edge processing through gateways contributes to enhancing the security and privacy of sensitive data. However, this approach also introduces new challenges, particularly in distributed systems, which are generally more vulnerable to attacks compared to centralized architectures [43]. Among the emerging challenges, the preservation of end-device location privacy stands out. In mobile scenarios, sensor nodes offload their data to the nearest gateways, making it possible to infer their location and movement patterns. Such exposure can be exploited by malicious actors, compromising user confidentiality and security [43].

## 9. Conclusions

This work proposed and evaluated the design of a low-cost dual-channel gateway targeting small- and medium-scale LoRaWAN networks, with a focus on mitigating the limitations associated with the use of the ALOHA protocol in single-channel gateways. The developed architecture incorporates multiple LoRa transceivers, including a dedicated unit for channel activity detection (CAD), enabling simultaneous reception on two distinct channels and a dedicated transceiver for CAD operations, without compromising the primary reception channels.

The case study, conducted in a real-world environment with the presence of coexisting LoRa networks, demonstrated performance gains through evaluation in three distinct scenarios. In the first scenario, which used static channel allocation without additional mechanisms, the dual-channel gateway achieved a PDR above 85% from SF9 onward. In the second scenario, with the introduction of CAD, a higher concentration of PDR values was observed, with results exceeding 92%, indicating a reduction in collisions and increased reliability. In the third scenario, which implemented dynamic channel switching based on timestamps, the dual-channel gateway achieved delivery rates above 94% for spread factors ranging from SF7 to SF12, consistently outperforming the single-channel gateway, whose PDR remained in the range 80% to 85%.

The experiments also demonstrated that increasing the SF contributes to greater signal robustness and an improved packet delivery rate. However, this benefit is associated with longer channel occupancy times and increased energy consumption, which are critical factors for battery-powered devices. In environments with coexisting LoRa networks, the proposed dual-channel gateway proved effective, particularly due to its capability to perform CAD before downlink transmissions. This mechanism reduces the risk of collisions with packets already in transit at different SFs, thus enhancing the reliability of the communication. Moreover, these features directly contribute to energy savings in end devices, especially those operating at higher SFs.

The results obtained validate the proposed solution as a technically and economically viable alternative to enhance scalability, reliability, and energy efficiency in IoT networks operating under budget constraints, particularly in urban or rural environments with a high density of devices. In summary, the proposed gateway architecture offers a promising alternative for small- and medium-scale IoT deployments. It balances performance and cost-effectiveness, improves reliability, and supports the evolution of more scalable and resilient LoRa networks.

As a future challenge, it is proposed to investigate the integration of the gateway with adaptive channel allocation algorithms and transmission coordination mechanisms with the aim of optimizing the medium access protocol. To this end, the exploration of CSMA- and TDMA-based approaches is envisioned, aiming to enhance network performance and efficiency in scenarios with coexisting networks. 

## Figures and Tables

**Table 1:** Comparison of studies on LoRa technologies and optimizations in communication networks.

Ref.	Hardware ou Software	Multiple Channels	Objective	Method/Approach	Benefits/Results
[34] (2019)	Software	Yes	Improve IoT gateways in terms of scalability, latency, and resource utilization.	Utilization of microservices and lightweight virtualization with Docker.	Distributed and modular processing at the network edge (edge computing).
[7] (2020)	Software	Yes	Optimize adaptive channel allocation in LoRa networks.	Adaptive allocation of multiple channels with different bandwidths.	Reduction in interference, retransmissions, and energy savings.
[20] (2021)	Hardware	No	Propose a multiprotocol gateway for IoT.	Integration of ZigBee and LoRa.	Interoperability between different protocols and performance optimization.
[35] (2022)	Software	Yes	Improve the scalability of LoRaWAN networks.	A new medium access control mechanism called Longest First Slotted CSMA (LFS-CSMA).	Optimization of channel utilization and reduction in collisions.
[9] (2023)	Software	Yes	Prevent collisions in dense networks.	Proposes a Collision Avoidance by Neighbor Listening (CANL) method that uses neighbor node listening.	Ongoing transmission monitoring and improved communication reliability.
[22] (2023)	Software	No	Evaluate the performance of unslotted ALOHA.	Analysis of the capture effect and multiple collisions.	Improved efficiency in multiple collision scenarios.
[4] (2024)	Software	Yes	Evaluation of collision avoidance algorithms.	Comparison between TDMA and CSMA.	TDMA demonstrates superior performance in collision prevention, while CSMA shows greater flexibility and scalability.
[21] (2024)	Software	No	Improve channel activity detection (CAD).	Enhance the scalability of LoRa networks by improving channel activity detection.	Reduction in collisions and better spectrum utilization.
[2] (2024)	Software	No	Development of a protocol with asynchronous downlink.	CSMA/CA with an asynchronous scheme for downlink.	Reduction in latency and increased network efficiency.
[8] (2024)	Software	Yes	Optimized channel selection in LoRaWAN networks.	Utilization of machine learning for optimized channel selection.	Reduction in interference and improved communication efficiency.

**Table 2:** Project Requirements.

Category	Requirement	Justification	Proposed Element for the Model
Hardware	Multiple simultaneous reception channels.	To reduce collisions and increase scalability in dense networks.	Multiple LoRa transceivers (SX126x).
Hardware	Capability to perform CAD without compromising packet reception.	Reduction in collisions during gateway-to-sensor node transmissions.	LoRa transceiver dedicated to CAD (SX126x).
Hardware	Time synchronization without Internet access.	To enable packet ordering and reception window control.	GPS (MAX-M10), RTC + TCXO (DS3231).
Hardware	Processing and network management unit.	Local processing for network management and decision-making.	ESP32-S3 (MPU), with support for TensorFlow Lite and ESP-DL.
Hardware	Expandable memory.	Support for expanding local data storage.	External FLASH and RAM memory.
Hardware	Wired and wireless network interface.	Flexibility for Ethernet or Wi-Fi connectivity.	Ethernet PHY e Wi-Fi.
Software	Compatibility with real-time operating systems.	Efficient management of concurrent tasks, interrupt control, and process prioritization in the gateway.	ESP32-S3 microcontroller, with native support for low-cost FreeRTOS.
Software	Support for embedded AI frameworks.	Local execution of lightweight AI models for real-time analysis and autonomous decision-making.	Vector acceleration unit of the ESP32-S3 for supporting AI libraries (TensorFlow Lite).

**Table 3:** Average CAD.

Devices	Scenario 2	Scenario 3
Node 01	1.4	1.3
Node 02	1.3	1.3
Node 03	1.3	2.0
Node 04	1.3	1.3
Node 05	1.4	1.4
Node 06	1.3	1.3
Node 07	1.4	1.4
Node 08	1.4	2.0
Node 09	1.4	2.0
Node 10	1.4	1.4
Node 11	1.3	1.3
Node 12	1.3	1.4
Node 13	1.3	1.3
Node 14	1.4	2.0
Node 15	1.3	1.3
Node 16	1.3	1.3
Node 17	1.3	2.0
Node 18	1.3	1.3
Node 19	1.3	1.4
Node 20	1.4	1.5
Average	1.34	1.51

## Data Availability

Data are contained within the article.
